# When Local Extinction and Colonization of River Fishes Can Be Predicted by Regional Occupancy: the Role of Spatial Scales

**DOI:** 10.1371/journal.pone.0084138

**Published:** 2013-12-18

**Authors:** Benjamin Bergerot, Bernard Hugueny, Jérôme Belliard

**Affiliations:** 1 Department of Hydrosystems and Bioprocesses Research Unit, Institut national de recherche en sciences et technologies pour l’environnement et l’agriculture, Antony, France; 2 Department of Technology, Architecture and Landscape, hepia Geneva, University of Applied Sciences Western Switzerland, Jussy, Switzerland; 3 Department of Biologie des Organismes et Ecosystèmes Aquatiques, Muséum National d’Histoire Naturelle, Paris, France; Texas Tech University, United States of America

## Abstract

**Background:**

Predicting which species are likely to go extinct is perhaps one of the most fundamental yet challenging tasks for conservation biologists. This is particularly relevant for freshwater ecosystems which tend to have the highest proportion of species threatened with extinction. According to metapopulation theories, local extinction and colonization rates of freshwater subpopulations can depend on the degree of regional occupancy, notably due to rescue effects. However, relationships between extinction, colonization, regional occupancy and the spatial scales at which they operate are currently poorly known.

**Methods:**

**And Findings**: We used a large dataset of freshwater fish annual censuses in 325 stream reaches to analyse how annual extinction/colonization rates of subpopulations depend on the regional occupancy of species. For this purpose, we modelled the regional occupancy of 34 fish species over the whole French river network and we tested how extinction/colonization rates could be predicted by regional occupancy described at five nested spatial scales. Results show that extinction and colonization rates depend on regional occupancy, revealing existence a rescue effect. We also find that these effects are scale dependent and their absolute contribution to colonization and extinction tends to decrease from river section to larger basin scales.

**Conclusions:**

In terms of management, we show that regional occupancy quantification allows the evaluation of local species extinction/colonization dynamics and reduction of local extinction risks for freshwater fish species implies the preservation of suitable habitats at both local and drainage basin scales.

## Introduction

The concept of metapopulation has gained a prominent role in both applied and fundamental ecology and emphasizes the importance of coupling local demographic processes with those occurring at regional scale, in particular dispersal [1]. According to the classic metapopulation model introduced by [2], a species persists within a landscape by a balance between local extinctions and colonizations from occupied sites within the region. In presence of such internal dispersal processes, the probability of colonization of an empty site is a positive function of the proportion of sites occupied because more sites produce more dispersers available for colonization [1]. In general, a positive link between colonization rate and regional occupancy is the signature of internal colonization (i.e. colonization from patches inside of the studied area), in combination or not with external colonization (i.e. colonization from sources outside of the studied area) [3]. Extinction rate per site could also be dependent on regional occupancy under the action of a process known as the rescue effect [4]. A high level of immigration contributes significantly to the increase of local population size and hence reduces the rate of local extinction. Therefore a small population on the verge of extinction could be rescued by a strong influx of immigrants. The rescue effect implies a negative relationship between colonization and extinction rates. If colonization rate is positively related to the fraction of occupied sites then extinction is negatively related to the fraction of occupied sites. For the sake of simplicity, let us define regional dependent rate of colonization or extinction as the increase (positive regional dependent rate) or decrease (negative regional dependent rate) of colonization or extinction rate when regional occupancy (i.e. the fraction of occupied sites) increases. With regard to regional dependent rate, different metapopulation types have been described [3,5] such as the Levins-like type (no regional dependent extinction rate, positive regional dependent colonization rate), the core-satellite type (negative regional dependent extinction rate, positive regional dependent colonization rate) or the island-mainland type (no regional dependent in both extinction and colonization rate). Therefore, regional effects on extinction and colonization rates are basic components of the theory of metapopulation, but are rarely quantified in the field (e.g. [6,7]) due to the difficulty of collecting relevant long-term data (to estimate colonization and extinction rates) over large areas (to estimate regional occupancy). It is even more difficult to gather such data for regionally co-occurring species to assess if they share the same type of metapopulation dynamics. Therefore, despite the importance of metapopulation theories in modern ecology and conservation biology, there is little information about the proportion of species that conform to particular spatial dynamics because few studies have examined species responses that occupy the same landscape [8].

Here we test the existence of regional effects on extinction and colonization rates in a river fish assemblage using an extensive dataset that covers the whole French territory (552,000 km^2^) and allows for computing annual local colonization and extinction rates per species and per site over periods of 8 to 22 years. A river is a branching network in which natural and anthropogenic barriers can easily reduce dispersal among habitats. Therefore, the metapopulation concept is an appealing theoretical framework to understand the dynamics of fish populations within a river network and, not surprisingly, there are a growing number of empirical studies that refer to it. This literature has been reviewed recently by [9] and, as expected due to the constraints imposed by data requirements, they listed few studies (e.g. [7,10]) assessing the importance of regional factors on extinction and colonization for entire assemblages. Clearly, our knowledge about the relationship between local extinction or colonization and regional occupancy is still poor for freshwater fishes. Moreover most of the studies reviewed by [9] did not quantify extinction or colonization rates at all, leading to the conclusion that general patterns of metapopulation dynamics are also poorly known. They also emphasized that the scales at which stream fish metapopulation processes operate are often unknown [9]. 

In this study we (1) quantify extinction rates and colonization rates for 34 French river fish species at local scale using annual fish community censuses performed on 325 sites in France, (2) predict regional occupancies using previously published species distribution models [11] and (3) assess whether local extinction and/or colonization rates of species differed among regions on the basis of the predicted proportion of occupied sites. Regional occupancy should display some variability to test its effect on extinction or colonization rates. In this regard our study differs from previous related ones [6,7] by focussing on the spatial variability displayed by the fraction of occupied sites among regions instead of considering its temporal variability within regions. We address the question of the spatial scale of metapopulation processes, in our case regional dependent extinction or colonization rates, by considering regions of different sizes, from river section to large hydrological units. We also account for differences among sites in their overall colonization/extinction rates to lower the blurring effect of local features. 

## Materials and Methods

### Ethics statement

All animal work was complied with the current laws of France and was approved by The French National Agency for Water and Aquatic Environments (ONEMA).

### Fish Data

**Figure 1:**
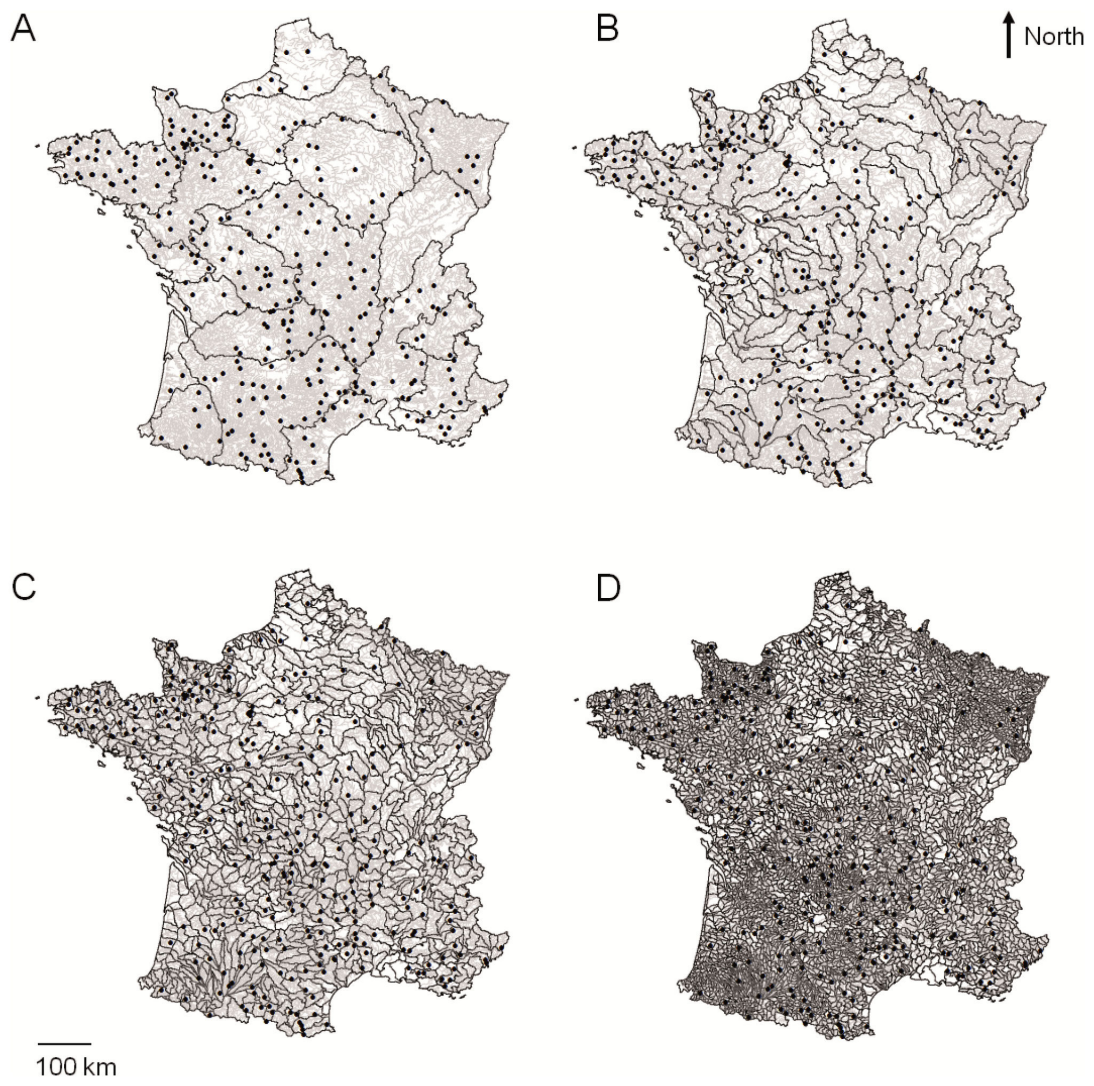
Map and location of the study sites. Map of France showing the 325 study sites (black points), main rivers (grey lines) and spatial scales used (A: hydrological unit scale, B: large-drainage basin scale, C: medium-drainage basin scale and D: small-drainage basin scale).

The dataset used in this study was provided by The French National Agency for Water and Aquatic Environments (ONEMA) and available on request at http://www.image.eaufrance.fr/divers/d-info-legale.htm. It included 325 sites distributed throughout France on small to medium size rivers (surface area of the drainage basin: 3 to 3756 km^2^) and annually sampled during at least 8 consecutive years (i.e. from 8 to 22 years) between 1979 and 2008 (Figure 1). Each site was entirely sampled by wading, using a double pass electrofishing method. This method enabled a realistic assessment of species presence/absence over the study period for each site [11,12]. Sites were long enough (129.69 ± 36.86 m) to include all available geomorphic habitat units (e.g. riffle, pools) and the home range of the dominant fish species [13,14] and therefore obtain a reliable picture of the species present (see 11,15 for further details). 

The database represents 3,800 standardized surveys (i.e. site – date combinations) and 3,140,977 fishes were caught, belonging to 59 species. Only 34 species were considered in analyses (Table 1). These are the most common species in France [11] and represented 98% of the individuals caught in our dataset. 

**Table 1:** List of species classified according to their family.

Family	Scientific name	Nsample	Pe (± SD)	Pc (± SD)	coefficients (Pe)	coefficients (Pc)
Anguillidae	*Anguilla anguilla*	190	0.24 (± 0.10)	0.49 (± 0.10)	0.22	0.16
Centrarchidae	*Lepomis gibbosus*	141	0.42 (± 0.13)	0.35 (± 0.09)	-0.16	0.15
Cobitidae	*Barbatula* spp.	251	0.08 (± 0.03)	0.60 (± 0.14)	-0.76*	0.34
Cottidae	*Cottus* spp.	189	0.11 (± 0.07)	0.45 (± 0.15)	-0.53	-0.01
Cyprinidae	*Abramis brama*	73	0.56 (± 0.14)	0.29 (± 0.08)	-0.50	-0.01
	*Alburnoides bipunctatus*	56	0.29 (± 0.16)	0.31 (± 0.12)	-0.50	-0.11
	*Alburnus alburnus*	109	0.33 (± 0.15)	0.40 (± 0.13)	-0.11	-0.26
	*Barbus barbus*	84	0.15 (± 0.08)	0.45 (± 0.14)	-0.55	0.18
	*Barbus meridionalis*	23	0.22 (± 0.12)	0.38 (± 0.11)	-0.33	0.10
	*Carassius* spp.	81	0.69 (± 0.12)	0.19 (± 0.03)	0.84*	-0.74
	*Chondrostoma nasus*	27	0.39 (± 0.14)	0.50 (± 0.14)	0.41	0.27
	*Parachondrostoma toxostoma*	38	0.47 (± 0.17)	0.32 (± 0.11)	0.01	0.17
	*Cyprinus carpio*	87	0.66 (± 0.12)	0.26 (± 0.06)	0.88**	-0.48
	*Gobio* spp.	234	0.12 (± 0.07)	0.38 (± 0.12)	-0.62	-0.27
	*Squalius cephalus*	207	0.12 (± 0.07)	0.40 (± 0.12)	-1.24***	0.05
	*Leuciscus* spp.	141	0.42 (± 0.15)	0.37 (± 0.11)	0.23	-0.04
	*Telestes souffia*	30	0.16 (± 0.08)	0.59 (± 0.10)	-1.04	0.78
	*Phoxinus* spp.	266	0.15 (± 0.09)	0.42 (± 0.13)	0.19	-0.4
	*Rhodeus amarus*	38	0.21 (± 0.08)	0.41 (± 0.12)	-1.21*	0.47
	*Rutilus rutilus*	200	0.25 (± 0.11)	0.47 (± 0.13)	-0.17	0.25
	*Scardinius erythrophthalmus*	131	0.72 (± 0.10)	0.22 (± 0.03)	1.00**	-0.45
	*Tinca tinca*	117	0.65 (± 0.12)	0.24 (± 0.05)	0.97**	-0.5
Esocidae	*Esox lucius*	104	0.54 (± 0.14)	0.3 (± 0.07)	0.77*	-0.19
Gadidae	*Lota lota*	8	0.45 (± 0.14)	0.23 (± 0.03)	-0.03	-0.47
Gasterosteidae	*Gasterosteus gymnurus*	45	0.56 (± 0.16)	0.31 (± 0.11)	0.45	-0.12
	*Pungitius laevis*	64	0.39 (± 0.14)	0.46 (± 0.14)	0.03	0.43
Ictaluridae	*Ameiurus melas*	56	0.50 (± 0.14)	0.32 (± 0.08)	0.36	-0.09
Percidae	*Gymnocephalus acerina*	24	0.43 (± 0.13)	0.28 (± 0.04)	-0.07	-0.18
	*Perca fluviatilis*	162	0.41 (± 0.12)	0.38 (± 0.09)	-0.10	0.19
	*Sander lucioperca*	34	0.78 (± 0.10)	0.19 (± 0.04)	1.17*	-0.3
Petromyzontidae	*Lampetra planeri*	153	0.27 (± 0.12)	0.51 (± 0.13)	-0.21	0.56
Salmonidae	*Salmo salar*	39	0.23 (± 0.09)	0.25 (± 0.07)	-0.6	-0.63
	*Salmo trutta*	289	0.17 (± 0.08)	0.42 (± 0.12)	0.45	-0.46
	*Thymallus thymallus*	19	0.32 (± 0.14)	0.23 (± 0.07)	-0.58	-0.43

Nsample: Number of sampling sites where species were recorded, Pe: Mean extinction probabilities, Pc: Mean colonization probabilities, SD: Standard Deviation, Pe and Pc: coefficient values associated to the modalities of the dummy variable species in the best extinction (respectively colonization) model (see Table 3). The asterisks show significant associated P-value (* p < 0.05, ** p < 0.01; *** p < 0.001).

Local extinction/colonization rates

Following [7], we calculated, for species i and site j, annual local extinction (Peij) and colonization (Pcij) probabilities as follows:

$$Peij=\frac{Number^{}of^{}times^{}the^{}site^{}was^{}occupied^{}at^{}time^{}{\left(t\right)}^{}and^{}unoccupied^{}at^{}time^{}(t+1)}{Number^{}of^{}times^{}the^{}site^{}was^{}occupied^{}at^{}time^{}(t)}$$

$$Pcij=\frac{Number^{}of^{}times^{}the^{}site^{}was^{}unoccupied^{}at^{}time^{}{\left(t\right)}^{}and^{}occupied^{}at^{}time^{}(t+1)}{Number^{}of^{}times^{}the^{}site^{}was^{}unoccupied^{}at^{}time^{}(t)}$$

The absence of a species in a sample may be due to a real absence but it is also possible that, because of sampling failure, the species was not detected even though it was present (pseudo-absence). Of course, this could potentially add a bias in colonization and extinction rates’ estimations. To overcome this problem, many methods for spatial sampling and spatial analyses exist (e.g. [16,17]). In this study, we have chosen to focus on electrofishing data with two passes, expecting that sampling effort is reliable to estimate true presence/absence of species. For a complementary data set including 171 sites sampled with similar sampling technique but with three successive passes, we found that species sampled at the third pass but not detected during the two first passes only concerned 2.5% of cases. Therefore, in average, the probability of detecting a species is very high (0.975). However it is likely that detection probability varies among species and/or sites and we cannot discard the possibility of value near zero for some species in some sites. If we consider the extreme case in which the variance in detection probability is due to a melange of two groups, one with perfect detection and a second with the lowest value compatible with the occurrence data, the correlation between observed and actual colonization or extinction is about 0.9 on the logit scale, a value which should be considered, given the assumptions made, as a lower bound. This high expected correlation between observed and actual extinction/colonization rates combined with a high sample size lead us to be confident in the ability of our analyses to find a meaningful relationship with regional occupancy if it exists. 

### Regional occupancy (RO)

Ideally, regional occupancy (RO) should be assessed from the observed occurrences of a species within a region by sampling all the localities or a representative set of them. In this study, the spatial coverage of the surveyed sites used in our study is not tight enough for this purpose. Therefore regional occupancy of a species has been calculated as the average value of the modelled probability of presence in each individual river segment of the region. In this aim, for each species, we coupled a model predicting presence probability (i.e. values potentially ranging from 0 to 1) as a function of local river features and climatic variables with a GIS-based segmentation of the entire river network of the French territory. These models’ predictions constituted the base of RO evaluation. For a given species, occupancy should differ among regions because of differences in the average habitat favourableness of the sites or in the climatic conditions. This spatial variability will be used to test the relationship between extinction/colonization rates and regional occupancy.

Species distribution models for each of the 34 species have been previously developed in France by [11,18]. These models, based on multiple logistic regressions, predict the probability of occurrence of species from one regional variable (belonging to one of eight hydrological units defined for the French territory which represent biogeographic constraints, [18]) and eight environmental site descriptors: slope (‰), elevation (m), July mean daily maximum air temperature (°C), January mean daily maximum air temperature (°C), stream width (m), mean depth (m), distance from headwater sources (km), and surface area of the drainage basin (km^2^). The subset of explanatory variables involved in each model varied according to fish species (see [11] for further details).

The explanatory variables of the models of [11] were calculated for continental France using available spatial environmental databases and a national hydrological network combined in a GIS-system [19]. The digital network was derived from a digital elevation model (DEM) with a spatial resolution of 50m provided by the French National Institute of Geography (IGN). The network comprised of ~115,000 uniquely identified segments that were defined by upstream and downstream confluences with tributaries and averaged 2.5 km in length. At each network segment, basin area, distance from the source, segment slope and elevation were derived from the DEM. Interannual mean monthly temperature variables were obtained by overlaying segment locations on grids of monthly temperature (resolution 1 km) that were interpolated from climatic data measured throughout France for the period 1961–1990. Hydraulic variables (segment wetted width and depth) were estimated at mean discharge using classical downstream hydraulic geometry relations (i.e. power laws relating mean segment depth and width to interannual mean discharge; [20]) whose coefficients were adapted to the French rivers using the hydraulic data described in [21,22]. Mean discharge of network segments was estimated using the models of [23], that interpolate observed average annual runoff for France (years 1981-2000). 

**Figure 2:**
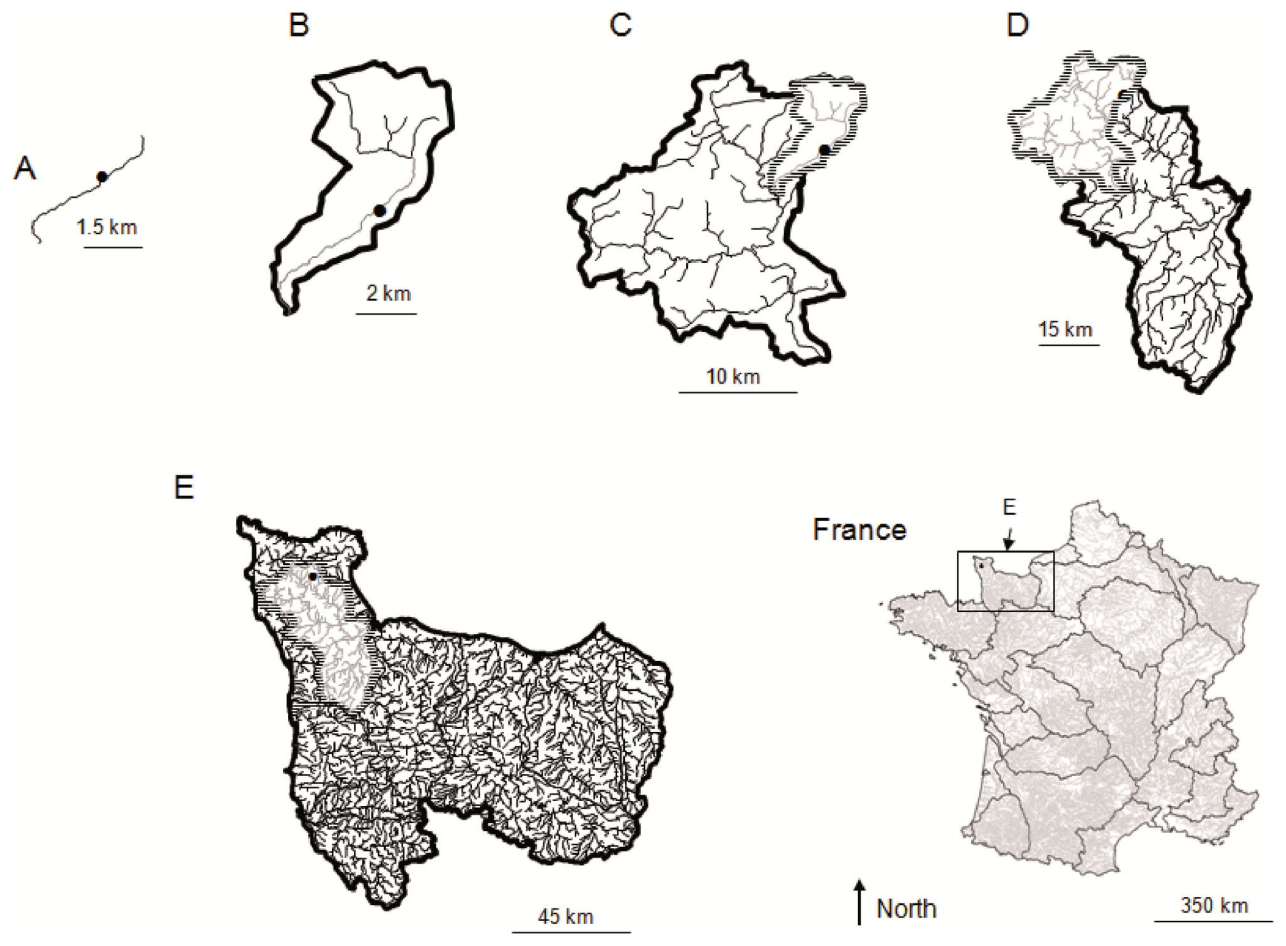
Regional occupancy calculation. Regional occupancy (RO) calculation for a given sampled site (black point) at different spatial scales (scale limits are represented by black bold lines). A. At the local scale, RO was given by the prediction of corresponding species distribution model for the river section supporting the sampled site (black line); B. At the small-drainage basin scale RO.2 was calculating by averaging RO.1 of the river sections composing this drainage unit (black lines). The river section supporting the sampled site (grey line) was excluded of the calculation to avoid any dependence of the RO estimation at the two spatial scales; C. At the medium-drainage scale, RO.3 was calculating by averaging RO.2 of the small-drainage basins composing this drainage unit (black lines), the small-drainage basin supporting the sampled site was excluded of the calculation (grey lines) and so on for D. large basin scale RO.4 and E. large hydrographical scale RO.5.

**Figure 3:**
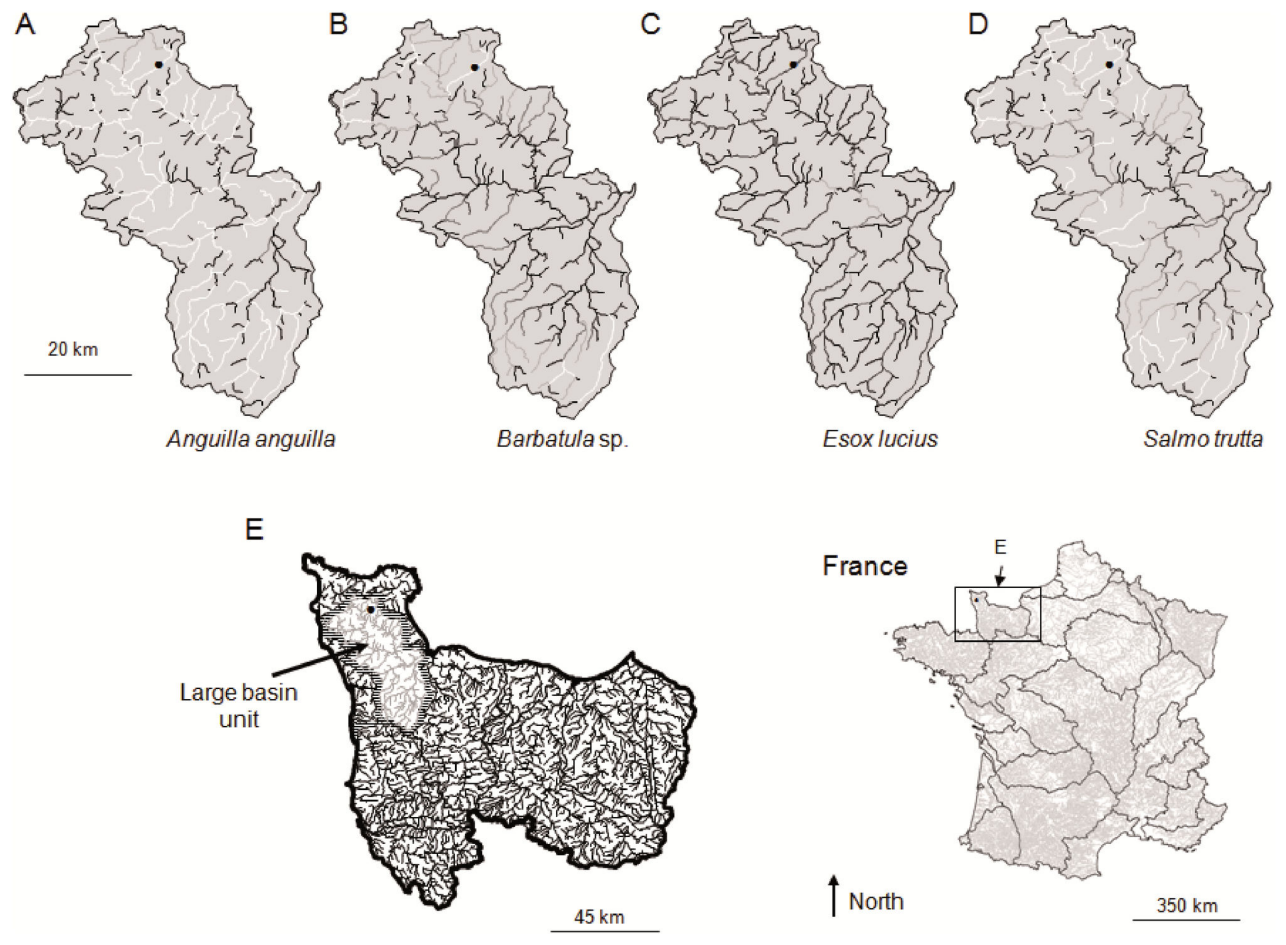
Examples of regional occupancy at river section scale for four species. Regional occupancy at river section scale (RO.1) for four species (A. *Anguilla anguilla*, B. *Barbatula* spp., C. *Esox lucius*, D. *Salmo trutta*) represented in large basin unit (E). From A. to D., white river sections represent high RO.1 value (near to 1) whereas black river sections represent low RO.1 value (near to 0). Black point represents a given sampled site.

For each sampling site i and each species j, regional occupancy ROij, was assessed at five independent nested spatial scales from “river section” to “large hydrographical” scale (Figure 2). In some way this pattern relates to the hierarchical physical framework defined by [24] to interpret stream ecological functioning, but we did not postulate any specific ecological process related to each of spatial scales. In fact the transition from one spatial scale to another was exclusively dictated by the river network structure and the occurrence of significant confluences. At the river section scale, ROij.1 was given by the prediction of the species distribution model for species i and for the network segment supporting the sampled site j (Figure 3); at the small-drainage basin scale, ROij.2 was calculated by averaging ROij.1 of the network segments included in the current drainage unit (the river section supporting the sampled site was excluded, so RO calculation at small-drainage basin scale was totally distinct from RO calculation at river section scale); at the medium-drainage basin scale, ROij.3 was calculated by averaging ROij.2 of the small-drainage basins included in the current drainage unit (the small drainage basin supporting the sampled site was excluded so RO calculation at medium-drainage basin scale was totally distinct from RO calculation at small-drainage basin scale) and so on for large-drainage basin scale (ROij.4) and large hydrographical unit scale (ROij.5). So, given the way of RO calculation (averaging species occurrence predictions), RO values potentially range between 0 and 1 whatever the spatial scale considered. For a given species, occupancy should differ among regions because of differences in the average habitat favorableness of the sites or in the climatic conditions. This spatial variability will be used to test the relationship between extinction/colonization rates and regional occupancy at various spatial scales.

It is worth noting that GIS-derived river segments are longer than the river reaches used to calibrate the species distribution models. Therefore the predicted value per segment should be interpreted as the expected fraction of sites occupied per segment under the hypothesis that sites within a segment are homogeneous with regard to the variables integrated into the model. Over a river length of about 2.5 km, all the variables but width and depth are unlikely to display great variability. Considering width and depth, our estimated values at the segment scale should be considered as an average of the site conditions over the river segment. Furthermore, because rivers segments are defined by two successive confluences, we hypothesize a sufficient homogeneity of both depth and width at this spatial scale. If the above assumptions hold true, we expect species distribution models to give similar results whether we use direct environmental measures for the 325 sample sites or environmental variables reconstructed for the corresponding river segments. We found a high correlation (0.85) suggesting that the difference in spatial scale between segment and site is not a source of concern.

Analysis

To increase the power of the analyses, all the sites-species combinations have been considered jointly according to the following models:

$$\log it(Pij)=Ei+Sj+\sum_{k=1}^{n}ROijk$$

where P_ij_ represents the probability of extinction/colonization for the i^th^ species and j^th^ site, E the dummy site variable, S the dummy species variable, k the spatial scale of ROij varying from k=1 to k=n, with n between 1 and 5 according to the considered model. By testing models with increasing values in n, we infer the spatial scale at which regional occupancy plays a role. E and S have been introduced to control for differences among species and among sites in extinction and colonization rates. For instance species may differ in extinction probability because they differ in population density [25]. Populations in sites exposed to high environmental variability are expected to have a lower lifetime than populations in more stable environments [1].

Data have been fitted with General Linear Models (GLMs) assuming binomial distribution of the extinction/colonization probability. For both extinction and colonization rates, six models have been fitted: one null model (no regional effect) and five models including a regional effect evaluated at an increasing spatial scale. The Akaike Information Criterion, AIC [26] has been used to select the best model. If there is a positive relationship between colonization rate and regional occupancy, the selected model should include one or several RO’s, all with positive regression coefficients. For extinction the same is expected but regressions coefficients should be negative. Then, analyses of variance of the GLMs were made using a type 3 ANOVA and associated P-values were calculated. The contribution for each independent variable in the best model fitted was calculated by applying the hierarchical partitioning algorithm [27].

Statistical analyses were performed with R^©^ version 2.15.1 (2012-06-22).

## Results

Among the 34 studied species, belonging to 12 families (Table 1 and Figure 1), brown trout (*Salmo trutta*), minnow (*Phoxinus* spp.), stone loach (*Barbatula* spp.) and gudgeon (*Gobio* spp.) were the most common species (i.e. sampled at least once in >70% of the sites). Conversely, the burbot (*Lota lota*), Mediterranean barbel (*Barbus meridionalis*), grayling (*Thymallus thymallus*) were the most scarce ones.

Pike perch (*Sander lucioperca*) and rudd (*Scardinius erythrophthalmus*) had the highest mean extinction probabilities and the lowest mean colonization probabilities. Conversely, stone loach had large colonization probabilities and low extinction probabilities. European eel (*Anguilla Anguilla*), European brook lamprey (*Lampetra planeri*) and riffle dace (*Telestes souffia*) presented the highest colonization probabilities. 

Local extinction and colonization by sites and species were significantly negatively linked (r=-0.56, p<0.001). Extinction and colonization were, respectively, negatively (r=-0.48, p<0.001) and positively (r=0.33, p<0.001) related to average occurrences of species. 

The best model explaining extinction/colonization of fish species included RO at all landscape scales with a species effect and site effect (Table 2). In this model, extinction was significantly negatively related to RO at river section (RO.1) and small-drainage basin (RO.2) scales (Table 3). RO at river section (RO.1) and small-drainage basin (RO.2) scale explained respectively 20.29%, 13.06% of extinction probabilities. Colonization was significantly positively related to RO at river section (RO.1, Table 3). RO at river section (RO.1) explained 27.04% of the total variance of colonization probabilities. At medium-drainage basin (RO.3), large-drainage basin (RO.4) and large hydrographical unit (RO.5) scales, extinction and colonization were not significantly related to species RO (Table 3). 

**Table 2:** Models tested using the GLM procedure and ordered following there associated Akaike Information Criteria (AIC) obtained by stepwise selection procedure.

**Models tested in GLM procedure for extinction**	**AIC**
Pe ~ Site + Species	3986
Pe ~ RO.1 + Site + Species	3822
Pe ~ RO.1 + RO.2 + Site + Species	3570
Pe ~ RO.1 + RO.2 + RO.3 + Site + Species	3541
Pe ~ RO.1 + RO.2 + RO.3 + RO.4 + Site + Species	3534
Pe ~ RO.1 + RO.2 + RO.3 + RO.4 + RO.5 + Site + Species	2929
**Models tested in GLM procedure for colonization**	**AIC**
Pc ~ Site + Species	3060
Pc ~ RO.1 + Site + Species	3015
Pc ~ RO.1 + RO.2 + Site + Species	2847
Pc ~ RO.1 + RO.2 + RO.3 + Site + Species	2832
Pc ~ RO.1 + RO.2 + RO.3 + RO.4 + Site + Species	2821
Pc ~ RO.1 + RO.2 + RO.3 + RO.4 + RO.5 + Site + Species	2375

Pe: probability of extinction, Pc: probability of colonization, RO.1: RO at river section scale, RO.2: RO at small-drainage basin scale, RO.3: RO at medium basin scale, RO.4: RO at large basin scale and RO.5: RO at hydrographical scale.

**Table 3:** Results of the best GLM model selected by Akaike Information Criterion between extinction/colonization and regional occupancy variables, species and site.

		Extinction			Colonization		
Variables	*Df*	*α*	*LR Chisq*	*p*	*α*	*LR Chisq*	*p*
RO at river section scale (RO.1)	1	-1.94	31.27	<0.001	1.13	9.59	<0.01
RO at small-drainage basin scale (RO.2)	1	-1.48	11.01	<0.001	0.73	2.41	0.12
RO at medium basin scale (RO.3)	1	-0.34	0.37	0.54	-0.14	0.06	0.81
RO at large basin scale (RO.4)	1	-0.33	0.33	0.56	0.46	0.55	0.45
RO at hydrographical scale (RO.5)	1	0.34	0.56	0.45	-0.48	0.93	0.33
Species effect	33	Table 1	154.11	<0.001	Table 1	55.84	<0.01
Site effect	245/232		188.00	0.99		138.33	0.99

RO: regional occupancy, Df: degrees of freedom, LR Chisq: Likelihood-ratio Chi-square test, α: estimate value of the model, p: the associated P-value.

**Figure 4:**
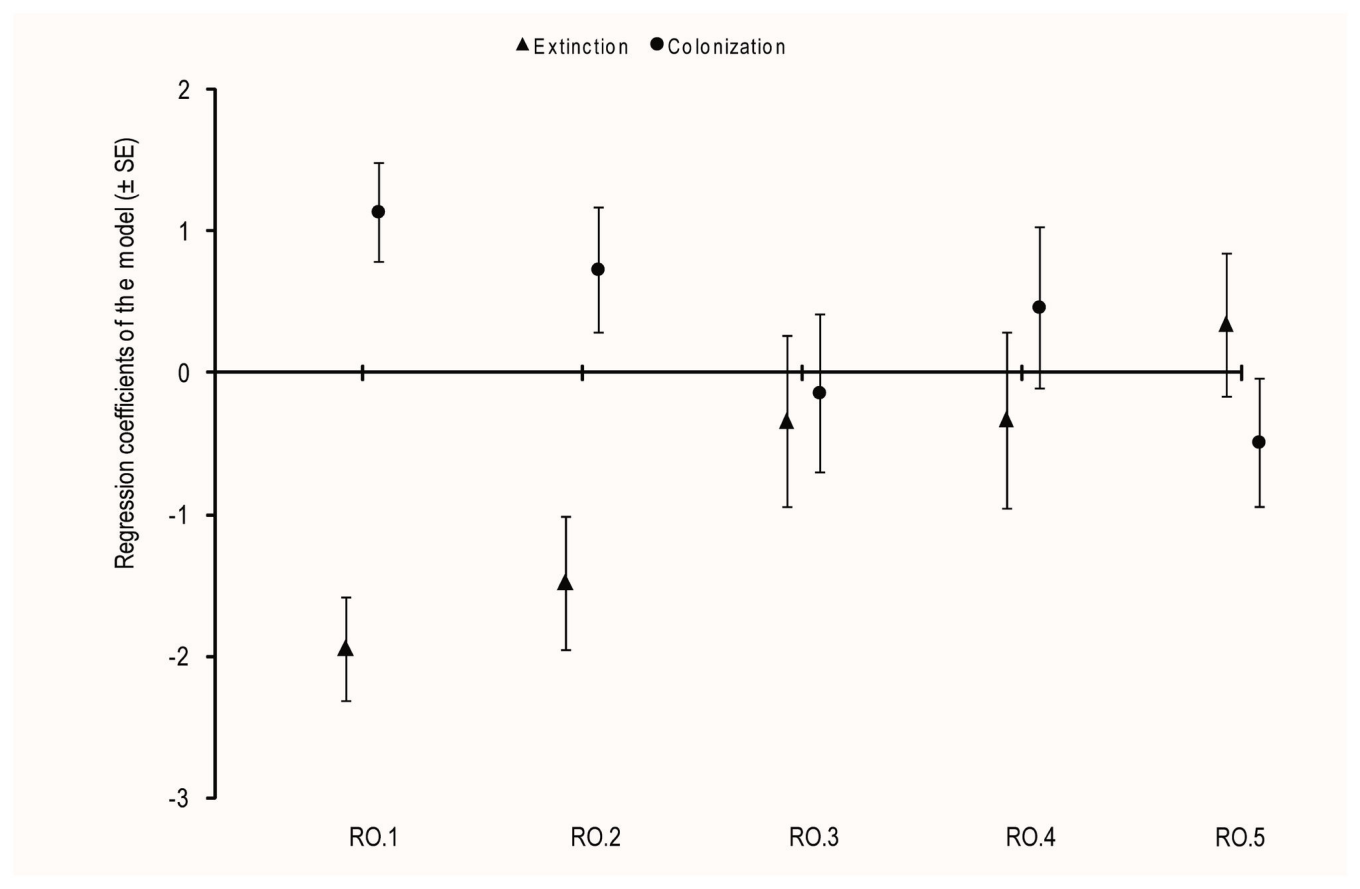
Extinction rates, colonization rates and regression coefficients. Regression coefficients between RO at different scales and extinction (black triangles) or colonization (black points) rates obtained by the best GLM model selected (± standard errors) in Table 3 (RO.1: RO at river section scale, RO.2: RO at small-drainage basin scale, RO.3: RO at medium basin scale, RO.4: RO at large basin scale and RO.5: RO at hydrographical scale).

The best extinction and colonization models showed that the bigger the spatial scale, the lower was the RO coefficients (Figure 4)

## Discussion

In this study, we analysed the relationship between local extinction and colonization rates of 34 common freshwater fish species and regional occupancy (RO) at various landscape scales. We showed that local extinction and colonization were respectively negatively and positively related to RO at river section (RO.1) and local extinction negatively related to small-drainage basin scales (RO.2). We also showed that the magnitude of the regional dependency (RO’s regression coefficients) is scale dependent and its absolute contribution to colonization or extinction rates decreases with distance. However at some larger spatial scales, we observed signs of regression coefficient between extinction/colonization and RO that were opposite, albeit small and non-significant, to the expectation.

To our knowledge, multi-species assessment of regional dependent colonization and/or extinction rates has been done only in two studies, both dealing with fish assemblages in river system [7] and in tide pools [6]. In contrast with our results, in both studies, weak relationships between colonization/extinction and regional occupancy have been found, suggesting that most species conformed to a metapopulation dynamic close to the island-mainland type (i.e. a constant probability of extinction/colonization, independently of regional occurrence). As the pioneering study conducted by [7] dealt with similar river fishes, it deserves to be discussed in details. Three factors may explain why no consistent effect of regional occupancy has been found in [7]. First, the size of the region that has been considered (a 950 km long river section) is probably too large with regard to most fish dispersal ability. Second, surveyed sites have not been an exhaustive sample of suitable sites and, in addition to low sample size (10 sites) this sampling scheme may have provided an uncertain estimation of the actual regional occupancy. Third, the influence of regional occupancy may have been blurred by differences among sites in their extinction and colonization rates, as these rates were related to the position in the river gradient. Our study circumvented those limitations. Indeed, different spatial scales for the region have been considered independently, an estimate of the occupancy over the entire region is provided and a site effect on extinction and colonization rates has been accounted for. The two studies differ in one important point; in [7], regional effects have been tested with regard to occupancy that varied through time within the same region, while in the present study, occupancy varies among regions and temporal variations are not accounted for. Our approach allows dealing with a greater sample size of regional occupancies and therefore is potentially more powerful.

Most metapopulation models with an explicit treatment of space assume that colonization rate of an empty site depends on the total number of immigrants produced by the region and that the contribution of a colonization source decreases with its distance from the focal site (e.g. [28]). Our results provide support to both these basic assumptions for river fishes. This is not surprising considering that dispersal is constrained by the river network and because river catchments are quasi-closed systems for freshwater organisms, so colonization is obligatorily mostly internal. However, to our knowledge it is the first time that such a conclusion is reached by studying colonization rates directly. Other studies emphasizing the role of internal colonization for freshwater fish were based on negative relationships between species richness (e.g. [29]), or single species incidence (e.g. [30]), and distance to colonization sources. Unexpectedly, we sometimes observed negative signs of regression coefficients between colonization rate and regional occupancy defined at large scale, but the associated effect were weak and non-significant. We have no explanation for these results besides considering them as a statistical noise. 

Our results also suggest that rescue effect is a major component of river fish metapopulation dynamic in our study system. Rescue effect occurs when a population is prevented from being extinct by the arrival of immigrants. It means that immigration rate should be sufficiently high to affect local population dynamics [1]. For sites of similar size, in situ defaunation experiments have shown that some species may recover half their original population size in less than one year [31]. Population size per site and the total number of immigrants that could be accumulated within a year are therefore of the same order of magnitude, a situation that offers a great potential for the occurrence of a rescue effect. If these results hold true for French river fishes, then, the occurrence of a rescue effect is a very plausible assumption. By comparing historical and recent distributional data in the Colorado River [32], reported a negative relationship between the probability of local extirpation and the historical number of occurrences among an assemblage of 25 river fish species. This result is compatible with a rescue effect but not necessarily. Indeed, the time interval used by [32] to assess extinction is so large (about 80 years) that the succession of several extinction and colonization events is likely. A species with a high occurrence may display a low apparent extinction rate due to a higher likelihood that a colonization will succeed to an extinction, a process that is not a rescue effect per se. 

**Figure 5:**
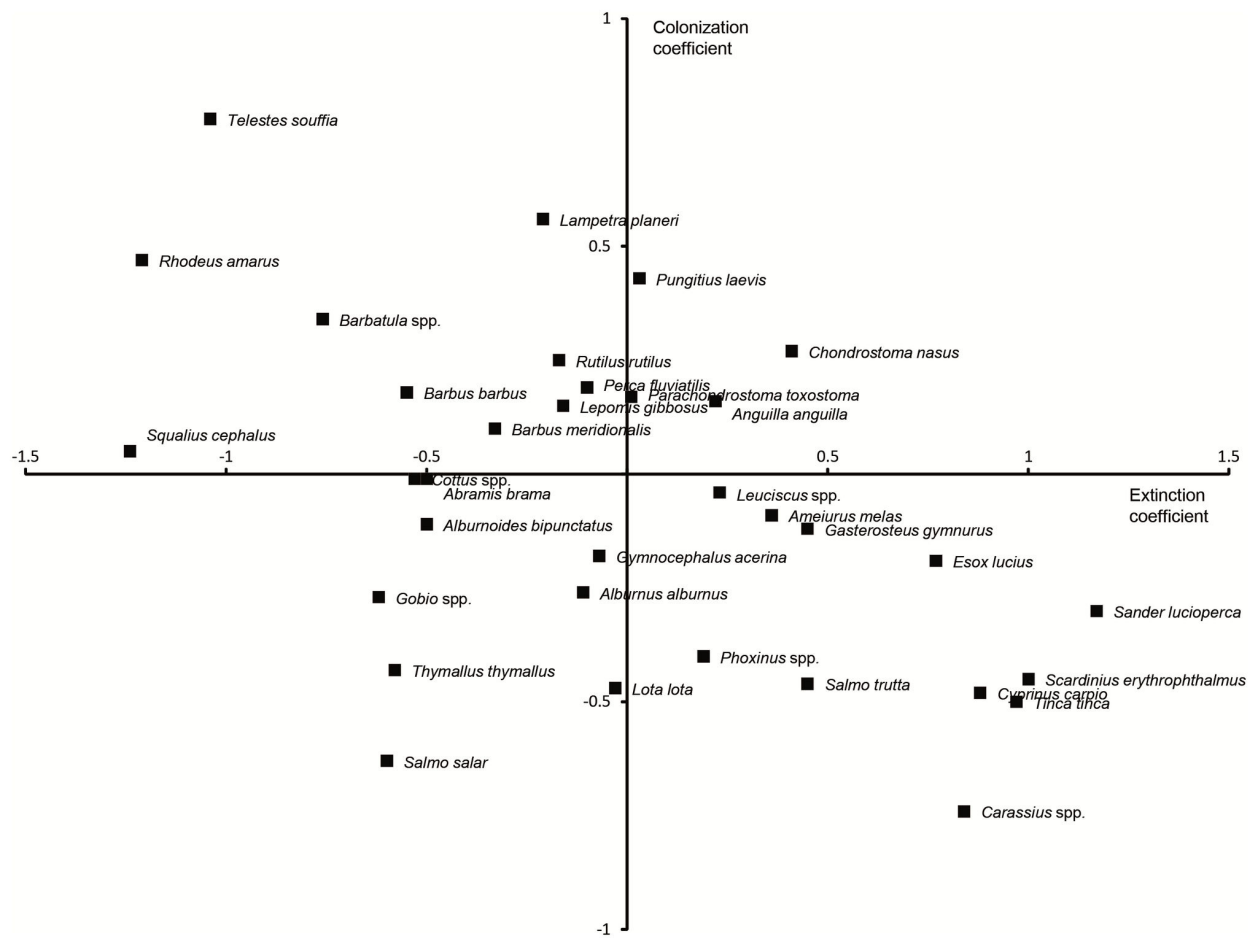
Ranking of species according to extinction and colonization coefficients. Relationship between species coefficient values from the best extinction and colonization models (see Tables 1 and 3).

In presence of a rescue effect, species with high colonization rates should also have low extinction rates, and, among species, colonization and extinction rates are expected to be negatively related. Such relationship is observed in our study (Figure 5). A metapopulation maintains itself as long as colonization rate is sufficiently high with regard to extinction rate. Therefore, species having intrinsically high colonization ability and low extinction risk are more likely to maintain balanced metapopulation dynamics in face of human induced decrease/increase of colonization/extinction rates than species having the opposites features. So we expect species with declining frequency of occurrence to have low extinction coefficient and/or high colonization coefficient (e.g. *Tinca tinca*). 

Using a larger dataset than ours [33], examined abundance time trends in French freshwater fish populations. It is interesting to note that our results are globally coherent with the species ranking established by [33] relative to their decreasing or increasing abundance. Indeed, independently of RO, species with high extinction coefficient (i.e. species most prone to local extinctions) generally exhibited a global decrease in their populations (e.g. *Tinca tinca, Salmo trutta* or *Esox lucius*), whereas species with both low extinction coefficients and high colonization coefficients (i.e. species least prone to local extinctions but most predisposed to colonization) exhibited mostly increasing populations (e.g. *Barbatula* spp. or *Rhodeus amarus*) [33].

However other processes, not implying extinction, may account for the observed relationship. One is the dependency of both extinction and colonization rates on population density. Indeed, local abundance could be a strong predictor of immigration and extinction rates [28]. Species that maintained high local abundances tended to have high immigration rates and low extinction rates whereas species with low mean abundances had lower immigration rates and higher extinction rate [28]. Moreover, in case of a strong rescue effect, some populations may persist by virtue of high immigration rates in sites that do not allow self-sustaining populations because of their poor habitat quality. As in all non-experimental studies, our results could be confounded by the action of an environmental variable correlated to both local colonization/extinction rate and regional occupancy. This could happen if an environmental factor displays spatial autocorrelation and is correlated to extinction or colonization rate leading those variables to be spatially autocorrelated. As regional occupancy is likely to be correlated positively or negatively with regionally averaged colonization or extinction rates, respectively, this could result in correlations between extinction/colonization rate and regional occupancy. The relationships observed between colonization/extinction rates and regional occupancy are compatible with metapopulation dynamics but further studies are needed to assess the importance of environmental spatial correlation.

The scales at which river fish metapopulations operate are often unknown [9], and our study is a first attempt to fill this gap for a representative assemblage of European fishes. The significant relation between extinction probabilities and RO at small-drainage basin scale (RO.2) can be explained because for most stream fishes intermediate spatial (and temporal) scales represent "the nexus" [34], where many important ecological and demographic processes occur. Indeed, persistence of species may have been greater in connected rivers because fish can move among adjacent habitats daily or seasonally for feeding, or to escape predation, to avoid unfavourable conditions, or to complete various life-history stages. Fish can migrate into connected basins to find better habitats when conditions deteriorate or rivers run dry [35]. In this context, it is important to note that medium-drainage basin or larger scale units referenced in this study may show major discontinuity (e.g. coastal zones in the North and the West of France separated by sea). That may explain the lack of significance between extinction and colonization probabilities at local scale and RO at larger scales. In our study, river section and small-drainage basin effects suggest that individual movements of fish occur mainly at reduced spatial scales and rapid recovery at larger spatial scale is limited. This is consistent with results from [29] (from three Illinois drainage basins) and [36], (from wadeable streams of eastern North America) suggesting that colonization processes of freshwater fishes occurred preferentially at the small-drainage spatial scale.

Environmental parameters used in this study did not take into account explicitly human pressures. Our models just explained 33.35% and 27.04% of extinction and colonization respectively and we suspect that a meaningful part of this unexplained variance could be linked to anthropogenic factors. So integration of human pressures in the calculation could provide valuable information to improve RO estimations in the future, notably because extinction and colonization processes could be influenced by human activities [31,37,38]. In particular, weirs and small dams, which are numerous along French rivers, could obfuscate the analysis of RO effect in small basins (RO.2), and partly explain the weak effect of RO at larger scales (RO.3, RO.4 and RO.5). In that way, human pressures could lead to over estimation of extinction rates compared to natural situations (possible influence of human pressure on colonization is much more unclear and could be probably positive or negative, depending on pressure type). However, in this study, of human pressure on colonization and extinction could be homogeneous because site effect for both colonization and extinction probabilities was not significant. Moreover, significant species effect for both extinction and colonization probabilities suggested that, independently of environmental conditions, intrinsic factors such as life-history traits or displacement ability [39] could favor (or limit) local extinction and colonization.

## Conclusions

To conclude, our results show that extinction/colonization risk evaluations can be made using RO estimations at different spatial scales. Our study reinforces the existence of metapopulation processes for freshwater fish but also allows the definition of the scale at which colonization and extinction processes could be comprehend in rivers. In conservation viewpoint, our results suggest that to reduce extinction risks of freshwater fishes at local scale, a good RO at river section scale (RO.1) is necessary and conservation and/or improvement of RO at small-drainage basin scale (RO.2) will be required to further reduce extinction risks. That means that localized habitat destruction may have regional consequences by lowering the amount of dispersers. Moreover, improving local habitat could be a waste of time if the existence of valuable habitats and populations were neglected at larger spatial scales (RO.2). In that sense, our results corroborate assumptions made by [40] which explained that local habitat heterogeneity restoration does not necessarily lead to higher biological diversity, particularly if dispersion from population sources is not guaranteed. Indeed, a more comprehensive understanding at which spatial scale extinction and colonization processes occur is needed [41]. Our study shows that the small-drainage basin scale seems to be the best scale to define conservation management plans for fishes and larger scales may be neglected, at least when considering species extinction risks at low temporal scale.

Finally, if predicting which species are likely to go extinct is perhaps one of the most fundamental yet challenging endeavours of conservation biology [37], direct estimation of extinction and colonization processes are frequently unattainable because of the lack of relevant data. To overcome this issue, our study provides new insights in the use of fish species distribution models (at the basis of our calculation of species regional occupancy) as a surrogate of extinction and colonization probabilities in species conservation programs. Moreover, our study shows inter-specific variability according to extinction and colonization. Indeed, independently of habitat factors, species are more or less able to colonize sites and more or less concern by extinction. To fully understand these inter-specific variations, intrinsic factors need to be studied into detail.”
